# Electroacupuncture for Tinnitus: A Systematic Review

**DOI:** 10.1371/journal.pone.0150600

**Published:** 2016-03-03

**Authors:** Miao He, Xinrong Li, Yang Liu, Juan Zhong, Luyun Jiang, Ying Liu, Qing Chen, Yan Xie, Qinxiu Zhang

**Affiliations:** 1 Chengdu University of Traditional Chinese Medicine, Chengdu, Sichuan Province, China; 2 Department of Otorhinolaryngology, Head and Neck Surgery of the Teaching Hospital of Chengdu University of Traditional Chinese Medicine, Chengdu, Sichuan Province, China; Johns Hopkins Bloomberg School of Public Health, UNITED STATES

## Abstract

**Background:**

Treatment effects of electroacupuncture for patients with subjective tinnitus has yet to be clarified.

**Objectives:**

To assess the effect of electroacupuncutre for alleviating the symptoms of subjective tinnitus.

**Methods:**

Extensive literature searches were carried out in three English and four Chinese databases (PubMed, EMBASE, Cochrane Library, CNKI, Wanfang Chinese Digital Periodical and Conference Database, VIP, and ChiCTR).The date of the most recent search was 1 June 2014. Randomized controlled trials (RCTs) or quasi-RCTs were included. The titles, abstracts, and keywords of all records were reviewed by two authors independently. The data were collected and extracted by three authors. The risk of bias in the trials was assessed in accordance with the Cochrane Handbook, version 5.1.0. (http://www.handbook.cochrane.org). Eighty-nine studies were retrieved. After discarding 84 articles, five studies with 322 participants were identified. Assessment of the methodological quality of the studies identified weaknesses in all five studies. All studies were judged as having a high risk of selection and performance bias. The attrition bias was high in four studies. Incompleteness bias was low in all studies. Reporting bias was unclear in all studies. Because of the limited number of trials included and the various types of interventions and outcomes, we were unable to conduct pooled analyses.

**Conclusions:**

Due to the poor methodological quality of the primary studies and the small sample sizes, no convincing evidence that electroacupuncture is beneficial for treating tinnitus could be found. There is an urgent need for more high-quality trials with large sample sizes for the investigation of electroacupuncture treatment for tinnitus.

## Introduction

Subjective tinnitus is a spontaneous, internally generated noise that could be described as the experience of a sound in the ear or in the head. The exact cause of subjective tinnitus remains unknown although in some cases it may be caused by a malfunction of the auditory end-organ, which in turn can be caused by several conditions. However, the commonest cause is age-related degeneration. Other causes are Meniere’s disease, trauma (acoustic or chemical), and cardiovascular diseases. The reasons for the onset of tinnitus remain unclear, and this may explain why different treatments for the disease are beneficial in some patients but not in others [1]. It is very common for patients with tinnitus to be very disturbed by the persistent noise and the impairment of their quality of life (QOL) and work, especially, patients with moderate/severe tinnitus.

Different treatment methods have been used to alleviate tinnitus, such as repetitive transcranial magnetic stimulation therapy [2–4], sound therapy (masking) [5],cognitive behavioral therapy[6], tinnitus retraining therapy[7], and pharmacological treatments (antidepressants, anxiolytics, and night sedatives). Because it would seem that there are in fact different etiologies for tinnitus, responses vary very much to different therapies. Many of these treatment options for tinnitus are not universally effective. Furthermore due to the limitations of the evidence provided by most trials on tinnitus treatment, the efficacy of most interventions for tinnitus remains to be demonstrated conclusively [8].

As a complementary or alternative therapy for tinnitus, acupuncture has been recommended for alleviating the symptoms of subjective tinnitus in some patients [9, 10]. Through the insertion and manipulation of needles at acupoints of the body, acupuncture could influence the function of the olivocochlear nucleus and trigger action potentials to rebalance the neurophysiological system [11]. According to the theory of traditional Chinese medicine, the pathogenesis of tinnitus can be classified into two categories: excess or deficiency syndromes. Excess syndrome is always caused by an invasion by an exogenous pathogenic factor, stagnancy of the blood, flaring of viscera fire, or accumulation of phlegm-dampness. Deficiency syndrome is closely connected to the insufficiency of viscera. Acupuncture is considered to be able to adjust these skewed conditions and maintain a balance between yin and yang.

Electroacupuncture is a modification of conventional acupuncture in which electrical stimulation is administered through acupuncture needles. It is believed that electrical impulses could strengthen the stimulation by the needles at acupoints. Because the body tissue can be a kind of electrical conductor, it is suggested that electrical stimulation can generate directional movement of ions and eliminate polarization of the cell membrane. Changes in the distribution and concentration of the ions in the tissues might be key factors for treatment using electrical stimulation at points. Studies have shown that electrical stimulation of the promontory in patients with tinnitus might increase microcirculation in a part of the auditory pathways [12] and interfere with tinnitus-generating circuits such as the dorsal cochlear nucleus and the inferior colliculus [13]. Although it seems that it is rational to use electroacupuncture for tinnitus, further investigation in a series of clinical trials is required to draw a reliable conclusion about the effectiveness of this treatment.

We found that there two systematic reviews have investigated acupuncture for the treatment of tinnitus [14, 15], these reviews included four clinical trials with electroacupuncture as the active intervention [16–18] or mock electroacupuncture as a sham control treatment [19].However, the study designs of these four trials were not described in sufficient detail, and the risk of bias was not thoroughly analyzed. The reviews seemed to be unable to show the efficacy of electroacupuncture as a treatment for tinnitus. With the publication of a fair number of studies on electroacupuncture for tinnitus in recent years, it may be reasonable to undertake a systematic review with the aim of summarizing the evidence from all the available rigorous trials of this special alternative treatment for tinnitus.

Thus, the purpose of the present systematic review was to critically evaluate the current evidence from randomized controlled trials (RCTs) on the use of electroacupuncture in patients with tinnitus, by using the guidelines of the Cochrane Handbook, version 5.1.0.

## Methods

### Search Strategy

We performed a systematic search for published and unpublished RCTs. The languages of the trials were limited to English or Chinese, but there were no publication year or publication status restrictions. The date of the search was 1 June 2014.

#### Electronic Searches

Electronic searches were performed in PubMed(1992 to 1 July, 2014); EMBASE (Excerpta Medical Databases) (1992 to July, 2014); Cochrane Library(Issue 9 of 12, July, 2014); Google; Chinese Cochrane Centre's Controlled Trials Register (up to July, 2014); Wanfang Chinese Digital Periodical and Conference Database (1997 to July, 2014); China National Knowledge Infrastructure (CNKI) Database (1992 to July, 2014), and VIP Chinese Science and Technique Journals Database (1992 to July, 2014).The Chinese Clinical Trial Registry Center was also searched for ongoing trials.

Two sets of text terms were used. The first set included terms for electroacupuncture, and the second set included terms for tinnitus. All kinds of researches of electroacupuncture for tinnitus were searched. The search strategy was not limited to RCTs so that the reference lists of all the papers obtained could be manually searched entirely. The search strategy for PubMed is described below. Similar search terms were adopted for the other databases.

- #1 electrical acupuncture
- #2 electroacupuncture
- #3 (electric* AND (acupuncture OR needle OR acupoint OR point OR stimulat*))
- #4 #1~#3/OR
- #5 tinnitus
- #6 tinnit*
- #7 #5~#6/OR
- #8 #4 AND #7

#### Searching Other Resources

References listed at the end of the identified publications were scanned for additional trials, and we contacted authors by telephone or email for additional data, if necessary. Some other existing systematic reviews possibly relevant to this systematic review were also retrieved from the electronic databases mentioned above for additional trials.

### Study Types

Prospective RCTs of electroacupuncture versus a placebo regimen were included. We excluded case studies, case series, qualitative studies, un-controlled studies, and trials without randomization methods.

### Participants

Patients presenting with unilateral or bilateral subjective tinnitus (not necessarily associated with hearing loss) were included. We excluded patients with objective tinnitus, which might be caused by inflammatory problems of the middle ear, acoustic tumor, head trauma, or cerebral vascular events.

### Interventions

We compared electroacupuncture with conventional medicine or with other intervention regimens. The procedure for electroacupuncture is to insert the acupuncture needle as would normally be done, attain the qi reaction by hand manipulation, and then attach an electrode to the needle. A pulsating electrical current could be applicated to acupuncture needles by an electro-acupuncture stimulator as a means of stimulating the acupoints to provide continued stimulation. Trials of electroacupuncture serving as a concomitant treatment with other types of interventions versus just the “other types of interventions” were also included. The other types of interventions considered for control groups included:

- conventional medicine, including antidepressants, neuromodulators, and other drugs such as intratympanic steroid injections
- Chinese herbal medicine
- conventional medicine combined with Chinese herbal medicine
- other complementary therapies, such as: moxibustion, cupping and auricular press, point-injection therapy.

The intervention regimen might fall into one of the following categories:

1. electroacupuncture alone
2. electroacupuncture with medicine (conventional medicine, Chinese herbal medicine, or both)
3. electroacupuncture with other complementary therapies
4. electroacupuncture with medicines and other complementary therapies
5. Trials with electroacupuncture performed as a part of a complex intervention versus another type of regimen, such as electroacupuncture plus Chinese herbal medicine vs. acupuncture, were excluded.

Placebo, conventional medicine, or other interventions regimens were considered for control groups.

### Outcome Measures

Studies were required to include either the symptom severity or the relief of symptoms of tinnitus as outcome measures. Other clinically important outcomes included the total effectiveness rate, the influence on quality of daily life, and adverse events.

#### Primary Outcomes

Improvement in tinnitus severity and disability could be measured by the clinical efficacy/clinical cure rate, or validated tinnitus-specific questionnaires. There is no limitation of how long the treatment sessions should last, for the number of treatment sessions and the duration of treatment might vary very much. The outcomes could be measured simply at the end of treatment. Possible primary outcomes included the following:

1. Improvement in overall symptoms of tinnitus
2. Improvement in quality of life
3. Clinical efficacy or clinical cure rate

#### Secondary Outcomes

1. Improvement in tinnitus loudness (measured by a grading scale)
2. Improvement in annoyance and awareness of tinnitus
3. Changes in auditory threshold
4. Adverse events: sensation during electrical stimulation, hemorrhage or infection of the points, fainting during acupuncture treatment, and other side effects associated with electroacupuncture

### Study Selection, Data Extraction, and Quality Assessment

#### Study Selection

Two authors (MH and XL) reviewed the titles, abstracts, and keywords of all records,which were retrieved separately, to determine whether the studies met the inclusion criteria. If there was any disagreement, it was resolved by discussion.

#### Data Extraction and Management

Before data extraction, a standard extraction form was prepared. Raw data, such as details of the authors, publication information, design of the original trial, and some unpublished data, were independently extracted by three authors (MH, XL, and JZ).

#### Assessments of Risk of Bias

The risk of bias was assessed using the recommendations in the Cochrane Handbook for Systematic of Interventions (version5.1.0, http://www.handbook.cochrane.org). Three authors (MH, XL, and JZ) independently evaluated the risk of bias in the included studies according to the recommendations. All disagreements were resolved by discussion. The risk of bias can be categorized as follows.

1. Randomization sequence generation: an adequate randomization sequence should be generated by computer or random digits table.
2. Allocation concealment: the person enrolling the next study participant should be blind to the randomization schedule.
3. Blinding of participants or healthcare providers: lack of blinding could bias the results by affecting the compliance of the participants and the performance of interventions by the healthcare providers.
4. Detection bias: whether blinding of participants and personnel in a trial is sufficient or not is closely related to detection bias. However, assessment of detection bias should be made separately for different outcomes.
5. Incompleteness bias: a high proportion of missing outcome data is often assumed to be the main cause for incompleteness bias.
6. Reporting bias: reporting bias could be assessed from selective reporting of outcomes. By comparing the outcomes in the published report with those in the protocol, it is possible to determine whether or not outcome reporting is sufficiently complete.
7. Other bias: this includes bias caused by conflicts of interest or abnormal distribution of the participants.

### Data Analysis

#### Assessment of Heterogeneity

We tested for statistical heterogeneity by using the chi square and I^2^ tests. A low *P* value (or a large chi-square statistic relative to its degree of freedom) provides evidence of heterogeneity of intervention effects. And I^2^ describes the percentage of the variability in effect estimates that is due to heterogeneity rather than sampling error. A rough guide to thresholds for the interpretation of I^2^ could be: 30%-60% may present moderate heterogeneity, 50%-90% substantial heterogeneity, and 75%-100% considerable heterogeneity. If statistical heterogeneity was detected, we would conduct a subgroup analysis and identify the cause of the heterogeneity. When there is inconsistency in the direction of effect, it would seem to be rational not to do meta-analysis.

#### Data Analysis

We were to perform a meta-analysis if the data were similar enough.The measurements of treatment effect for dichotomous data were to be expressed as relative risks along with 95% confidence intervals (CIs); for continuous data, mean difference and 95% CIs would be adopted. The statistical significance of the hypothesis test was set at α = 0.05 (two-tailed z tests). If different types of interventions were used in a study, a subgroup analysis would be performed. Funnel plots were to be used to assess publication bias, if the number of included studies exceeded 10.

## Results

### Study Description

#### Search results

We retrieved 89 trials using the search strategy specified in our protocol. We discarded 73 articles after reviewing the titles and/or abstracts. Eleven studies which initially appeared to meet the inclusion criteria were excluded following assessment with the text. There was no unpublished or ongoing study. Thus, five studies finally met our criteria and were included in this review. The study selection process is outlined in S1 Fig.

#### Included Studies

Two studies employed mixed interventions that included electroacupuncture plus either Chinese herbal medicine and psychotherapy [20], or mixed administration of herbal and conventional medicines [21]. Another two studies compared the efficacy of electroacupuncture with that of manual acupuncture [22]or placebo acupuncture [23]. One study investigated the effect of electroacupuncture versus manual and placebo acupuncture simultaneously [24]. Most of the included studies were conducted after the year 2000. Three of the included studies were from China [20–22], and the other two were from England [23] and Denmark[24]. Four studies adopted a two-armed parallel group design [20–23]. One used a three-armed parallel group design [24]. In the three studies conducted in China [20–22], acupoints selected for the treatment were classified into either main or adjunct points. Three to four main acupoints were used. In each session, any two main points were selected for electrical stimulation alternatively. The adjunct points received only manual stimulation, both in the treatment group and the control group. In the trial conducted in Denmark [24], a total of 14 local and remote points were chosen for electrical or manual stimulation. Each treatment session lasted for 6 to 15 days, and there were 2 to 6 consecutive courses. When measuring the outcomes, three of the trials [20–22] used efficacy rate to assess the therapeutic effect of tinnitus, which were ranked into four degrees: cured, markedly effective, effective and ineffective. This criterion is mostly based on the improvement of the symptoms and the accompanying symptoms of tinnitus. One study [23] evaluated the efficacy of treatment with a verbal description by the patient, tinnitus matching and visual analogue scales of the loudness/severity of tinnitus. One trial [24] use the visual analogue scales to assess the improvement of tinnitus frequency, loudness and reduction in quality of life. The details of the five trials are listed in S1 Table.

#### Excluded Studies

We excluded 11 studies [25–35] (S2 Table).Two had no or unclear randomization [25, 30], five did not set a control group [26–27, 29, 33, 35] and four did not meet the inclusion criteria due to the incomparability of the interventions in the treatment and control groups [28, 31, 32, 34].

### Risk of Bias

#### Allocation (Selection Bias)

Four trials were designed as randomized controlled studies [20–22, 24]. In the study by Zhang (2002) [22], patients were allocated to treatment or control groups according to the visiting sequence. This method of allocating participants was not truly random, and this study could be defined as a quasi-randomized trial. There was a great risk of selection bias in this study because the allocation was not adequately concealed. In three RCTs [20, 21, 24], the method of randomization and allocation was not mentioned in detail. Because of the lack of allocation concealment and the other details of the randomization procedure, there might be a high risk of selection bias in these trials. The study conducted by Marks et al. (1984) [23] was a double-blind cross-over controlled trial. As a special type of RCT, cross-over studies should also involve proper randomization tools for the allocation of participants. However, in this study, the authors failed to clearly describe how the participants were allocated. So, there was a high risk of selection bias in this study.

#### Blinding (Performance Bias and Detection Bias)

In the study by Wang et al. (2010) [24], patients were blinded to the treatment condition. All the treatments were performed by the same experienced acupuncturist. Placebo acupuncture needles were used, which were half-cut with a blunt tip. The half-cut needle was inserted through a cube-shaped piece of elastic foam to obscure the patients’ vision of the insertion points. A pricking sensation could be felt when the blunt needle touched the skin. Although the patients perceived that the needle was inserted into their body, the skin remained un-punctured. Patients in the acupuncture or the placebo group could be blinded by this method. However, it is still unclear how the patients were blinded to the electroacupuncture and manual acupuncture methods. In the electroacupuncture group in this study, an electrical stimulator was connected to the needle when the sensation of Deqi was felt. It was not mentioned whether a mock electrical stimulator was used to confuse the patients in the manual acupuncture group. So, it was unclear whether the patients in the electro- and manual acupuncture groups were indeed blinded to the group. To provide placebo electrical stimulation, the needles in the controlled group could be connected to the electroacupuncture stimulator, which would deliver a soft sound and a light flash at a certain frequency but no current to the surface electrodes [14]. Marks et al. [23] used a double-blind crossover method to study 14 patients with tinnitus. Non-penetrating needles were used as the placebo treatment. In this method, points out of the patients’ line of vision were selected. The skin was penetrated by the needle, which was removed immediately without the patients’ knowledge. At the time the treatment ended, the procedure was covertly repeated to give the sensation of needle removal. In addition, to blind the outcome assessor, researchers who knew the whole course of treatment were not involved. In this way, the risk of performance bias and detection bias in this study was low. In the other three studies [20–22], blinding was not mentioned. Perhaps blinding was difficult because the materials and manipulations used in the treatment were totally different in the test group and the control group. However, at least the outcome assessors should have been blinded. Because there was no blinding in these studies, there might be a high risk of performance and detection bias.

#### Incomplete Outcome Data (Attrition Bias)

In the study by Wang et al. 2010 [24], the sample size was calculated. A total of 45 subjects were required to limit the risk of type I and type II errors to 5% and 20%. In fact, 60 patients were recruited and randomized to one of the three groups. When the whole process of treatment and follow-up for about 70 days had ended, 10 patients had withdrawn (16.67% withdrawal rate). Therefore, this study was at a low risk for incomplete outcome reporting bias. However, there were some limitations to this study in terms of incompleteness bias. First, the reasons why the 10 patients withdrew were not reported. Second, intention-to-treat analysis and per-protocol analysis were not conducted. In the other four studies [20–23], there were no sample size calculations, and no cases were reported to have been lost to follow-up or withdrawn from the trials. The incompleteness bias might be unclear in these trials due to no report of drop-outs.

#### Selective Reporting (Reporting Bias)

To lower reporting bias, all analyses with and without statistically significant differences should be reported. One of the ways to assess reporting bias is to compare the results in the final reports with those in the protocol. However, no protocol could be found in these studies, and none of these trials declared a clinical trial registration number. So, it was difficult to assess whether all the outcomes had been included in the published reports. The risk of reporting bias in these studies was classified as “unclear”.

#### Other Potential Sources of Bias

No other potential sources of bias were found.

Risks of bias of included studies were listed in S3 Table and reasons for risk assessment were listed in S4 Table.

### Effects of Interventions

Five trials (322 participants) [20–24] were included in this review. The types of interventions used in the included studies can be classified as follows.

#### Electroacupuncture vs. Acupuncture

One study tested the effectiveness of electroacupuncture on the symptoms of tinnitus, as compared with the effectiveness of manual penetrating acupuncture [22].

In the study by Zhang [22], dichotomous outcomes were used to describe the results. The chi-square test showed that the effectiveness rate was 81.82% in the electroacupuncture group and 62.5% in the acupuncture group, with a significant difference between the two groups. It was indicated that electroacupuncture was superior to manual acupuncture for the treatment of tinnitus.

#### Electroacupuncture vs. Placebo Acupuncture

The efficacy of electroacupuncture and placebo acupuncture was compared in the studies conducted by Marks et al. [23] and Wang et al. [24]. Wang et al. [24]used a cube-shaped piece of elastic foamto obscure the patients’ vision of the insertion points. The needle was half-cut and blunt. When the needle touched the skin, a pricking sensation could be felt, but the skin remained un-punctured. In this study, visual analogue scales were used to evaluate the frequency of tinnitus occurrence, tinnitus intensity, and reduction in the quality of daily life, and for a subjective general evaluation of the treatments. In addition, hearing improvements were shown by audiograms. The results were presented as continuous data. Analysis of variance and *t*-tests were used to statistically compare the data. They found that the frequency of tinnitus occurrence and tinnitus loudness were significantly decreased in the electroacupuncture group (*P*< 0.009). Quality of life was improved and 1 month after treatment, as compared with the baseline in both the manual acupuncture and electroacupuncture groups (*P*< 0.038), but there were no significant differences in the quality of life after treatment between the groups (*P*> 0.091). Audiograms did not show any significant differences after treatment between the groups. The overall subjective evaluation was significantly better in the electroacupuncture group than in the manual acupuncture group and the placebo group. The results indicated that there is no statistically significant difference in the efficacy of placebo acupuncture and electroacupuncture treatment for tinnitus; however, electroacupuncture did confer some relative advantages.

Marks et al. [23] also used non-penetrating acupuncture as the placebo treatment. Needles were used to prick the skin and were removed immediately without the patients’ knowledge. Patients were not informed about Deqi. Although the details of the duration of placebo treatment in the study by Marks et al. differed from those in the study by Wang et al., in both studies, the skin was not penetrated, and the patients did not know about the therapeutic group to which they belonged. Both studies found no statistically significant differences in the effects of electroacupuncture and placebo acupuncture on tinnitus. However, the study by Wang et al. [24] showed that electroacupuncture does confer some relative advantages in terms of the improvement in the quality of life and the overall subjective evaluation after treatment compared with the baseline data.

#### Electroacupuncture Plus Chinese Herbal Medicine and Psychotherapy vs. Herbal Medicine and Psychotherapy

In the study by Chen et al. (2013) [20], efficacy rate was used to evaluate the outcomes. The efficacy rates in the electroacupuncture group and the control group were 83.8% and 46.7% respectively, which demonstrated that the former treatment was more effective for alleviating the subjective symptoms of tinnitus.

#### Electroacupuncture Plus Chinese Medicine and Conventional Medicine vs. Medicine Only

Wang et al. (2013) [21] compared electroacupuncture plus medicines with medicines only. The drug therapy consisted of intravenous drips of lidocaine and puerarin and oral administration of flunarizine capsules. The dichotomous outcome of efficacy rate was reported. The total efficacy rate was 83.3% in the electroacupuncture group and 60.0% in the control group, with a statistically significant difference between the two groups. This demonstrated that electroacupuncture plus medicine was more advantageous than medicine alone.

## Disscusion

### Summary of Main Results

#### Overall Completeness and Applicability of Evidence

Few RCTs have tested the effectiveness of electroacupuncture on tinnitus. The limited evidence suggests that electroacupuncture is beneficial for tinnitus, as compared with manual acupuncture, medicine, or psychotherapy. However, it is difficult to generate reliable findings due to the small sample size and poor quality of these studies. Although there are many validated questionnaires to measure improvements in tinnitus severity and disability, none of the included trials used these questionnaires for outcome measurements. In most Chinese studies, the outcomes were reported using the effectiveness rate. According to Clinical Guideline for Tinnitus of China in 2007, the therapeutic effect of tinnitus could be ranked into four degrees: cured, markedly effective, effective and ineffective. This criterion is mostly based on the improvement of the symptoms and the accompanying symptoms of tinnitus. Although it is an obscure criterion to some degree, it has been used by quite a few trials in China. In recent decades, there are four questionnaires most frequently used to assess the severity of tinnitus as well as the efficacy of therapeutic interventions, such as lowa tinnitus handicap questionnaire (THQ), tinnitus reaction questionnaire (TRQ), tinnitus handicap inventory (THI) and tinnitus questionnaire (TQ). These questionnaires were found to correlate very well with the severity assessed [36]. And the THI was proved to yield excellent internal consistency reliability and significant correlations were found between the THI and the symptom rating scales [37].This questionnaire has been translated into Chinese to evaluate the impact of tinnitus on the quality of life of the patient. No matter what kind of subjective outcome measurement tool is used, the rationale behind reporting should be to provide the information needed to allow replication of a study, reduce ambiguity and enhance transparency.

Before our study, there have been three systematic reviews [14,15,38]that focused on the efficacy of acupuncture as a treatment for tinnitus. RCTs that compared any form of acupuncture with any control intervention for the treatment of tinnitus were included in these two studies. The review by Park et al. [14] included six RCTs, two of which involved the use of electroacupuncture. One of these RCTs was the study by Marks et al. (1984) [23], and the other was a study by Axelsson et al. (1994) [19]. In the latter study [19], mock electroacupuncture was used as a sham control treatment with classical Chinese needle acupuncture as the active treatment. During the placebo treatment with mock electroacupuncture, surface electrodes were placed on the same regions as the acupuncture needles and connected to an electroacupuncture stimulator, but there was no output from the stimulator to the electrodes, and no electrical stimuli were transmitted via the surface electrodes. Because the aim of our study is to investigate the effect of electroacupuncture on tinnitus, the study by Axelsson et al. [19] was excluded. In the review conducted by Kim et al. (2012) [15], a total of nine studies were included, two of which compared the effectiveness of acupuncture or electroacupuncture with sham acupuncture for treating tinnitus. These two studies [23, 24] were also included in our review. The latest systematic review of Liu [38] was finished about 2 years ago. Five trials were included[30, 39–42]. The study of Qin [30] was also analyzed in our manuscript. Podoshin et al [39] investigated the effect of three treatment modalities of idiopathic-subjective: acupuncture, biofeedback and Cinnarizine. Biofeedback is a process that enables individuals to learn how to change physiological activity for the prupose of improving health and performance, which doesn’t refer in particular to electroacupuncture, so we didn’t involve this study. We excluded the other three studies conducted by Ding [40], Wu [41]and Xia [42] because randomization was not mentioned, which could not meet our inclusion criteria.

Since electroacupuncture has been widely used for treating tinnitus in China in recent years, there might be an urgent need to update the systematic review to present an objective evaluation of this treatment.

### Quality of Evidence

The five included trials were randomized, prospective, placebo-controlled studies. However, none of the trials described the method of randomization. In one trial [23], the method of double-blinding was described clearly. In the trial by Wang et al. [24], participants were single-blinded for acupuncture and placebo-acupuncture therapy. The other three trials [20–22] did not state whether the design was double-blinded or not. There is, therefore, a potential risk of measurement and implementation bias. The placebo effect could be an important factor that might influence treatment outcomes, and a double-blinded design is important to avoid such an effect. It might be rational to use mock electroacupuncture as a placebo treatment when active electroacupuncture is the treatment under investigation.

None of the trials described allocation concealment.

It was not clear whether incomplete outcome data were adequately addressed. In the study by Wang et al. [24], the drop-out rate was ideal. The other four trials [20–23] did not report drop-outs. The risk of incomplete outcome data bias in these five studies was therefore unclear.

It was not known whether these five trials reported all the pre-specified, expected results. Therefore, the risk of selective reporting bias was unclear.

### Potential Biases in the Review Process

There was no ongoing trial of electroacupuncture for tinnitus. The conclusion of this review was drawn from the five trials, which had a limited number of participants. We hope to include more studies in future reviews.

### Agreements and Disagreements with Other Studies or Reviews

Although three new trials of electroacupuncture for tinnitus were included in our review, our results are similar to those of previous reviews[14,15].Due to the poor methodological quality of the primary studies and the small sample sizes, no sound evidence could be obtained to support electroacupuncture as a treatment for tinnitus.Further high-quality trials are still required.

## Authors’ Conclusion

There are insufficient quality data to recommend the routine use of electroacupuncture for subjective tinnitus. The benefit found in this systematic review could be due to publication bias and study-design limitations of the individual studies. In addition, the safety of electroacupuncture for subjective tinnitus is unknown due to the lack of adverse events reported.

### Implications for Practice

There is very limited support for the use of electroacupuncture for the treatment of tinnitus. Studies have suggested that electroacupuncture is effective for tinnitus in the short term; however, we found insufficient data to provide any support for the efficacy and safety of this treatment in the long term.

### Implications for Research

There is an urgent need of prospective, randomized, placebo-controlled, double-blind studies on electroacupuncture as a treatment for tinnitus. The outcomes should be evaluated using uniform, validated, tinnitus-specific questionnaires and uniform measurement scales. Specific stimulus parameters for electrical stimulators should be clarified. Long-term follow-up is necessary for the assessment of the long-term effectiveness and safety of electroacupuncture for tinnitus treatment.

## Supporting Information

S1 PRISMA Checklist (DOC)

S1 Fig Flow diagram of the study selection process in this systematic review. (TIF)

S1 Table Characteristics of the included studies. (DOCX)

S2 Table Reasons for exclusion of the 11 studies (DOCX)

S3 Table Risks of bias of included studies. (DOCX)

S4 Table Reasons for the risk of bias assessment. (DOCX)
