# Supplementary figures and images for: Electroacupuncture for Tinnitus: A Systematic Review
Source: PLoS One. 2016 Mar 3;11(3):e0150600. doi: 10.1371/journal.pone.0150600 (PMC4777560; doi:10.1371/journal.pone.0150600)

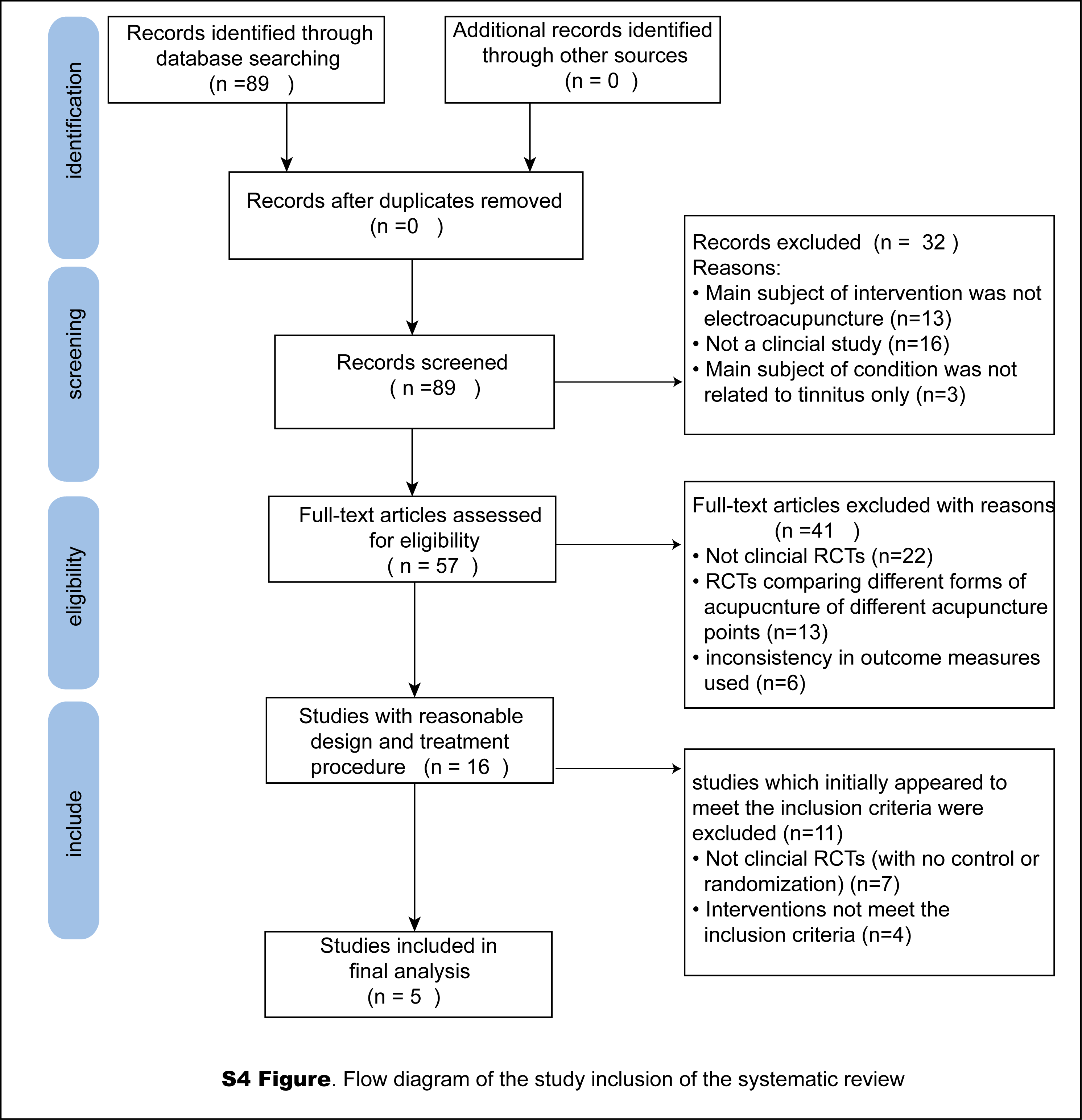

Supplement: S1 Fig — (TIF) [file pone.0150600.s002.tif]
