# Supplementary material for: Electroacupuncture for Tinnitus: A Systematic Review
Source: PLoS One. 2016 Mar 3;11(3):e0150600. doi: 10.1371/journal.pone.0150600 (PMC4777560; doi:10.1371/journal.pone.0150600)
Supplement: S1 Table — (DOCX) [file pone.0150600.s003.docx]

**S1 table. Characteristics of the included studies**

**Chen et al. (2013) [20]**

| Method | Randomized, prospective, controlled study |
| --- | --- |
| Participants | 60 patients with primary tinnitus  Gender: not mentioned  Age: 20–60 years (mean age not mentioned)  Tinnitus duration: 15 days to 10 years  Location of tinnitus: not mentioned  Audiography: not mentioned  Groups: treatment group, 30 patients; control group, 30 patients  Inclusion criteria: patients with primary tinnitus  Exclusion criteria: patients with tinnitus caused by disorders of the middle ear, intracranial neoplasms, or disorders of the nerves |
| Interventions | Treatment group:  Electroacupuncture (EA) was conducted at acupoints in the head and body. In each session, any two acupoints around the ears were chosen, such as Tinghui (GB2), Yifeng (SJ17), Ermen (SJ21), and Tinggong (SI19). Acupoints on the body were stimulated individually according to syndrome differentiation by traditional Chinese medicine (TCM). Each patient underwent 10 daily EA sessions for three consecutive courses. At the same time, patients also took herbal medicines based on syndrome differentiation, such as honeysuckle and forsythia powder for heat-wind of the lung, augmented rambling powder for flaming-up of liver fire, and clear the qi and transform phlegm pill for phlegm-fire. The herbal medicine was taken two times a day for three consecutive courses. Each course lasted for 10 days. In addition to EA and TCM, psychotherapy was also conducted to alleviate nerves and anxiety.  Control group:  Patients received only herbal medicines and psychotherapy. |
| Outcomes | Efficacy rate  According to Clinical Guideline for Tinnitus of China in 2007, the therapeutic effect of this disease could be ranked into four degrees: cured, markedly effective, effective and ineffective. This criterion is mostly based on the improvement of the symptoms and the accompanying symptoms of tinnitus. Although it is an obscure criteria to some degree, it has been used by quite a few trials in China. |
| Notes | Loss to follow-up/withdrawal/drop-out rates and adverse events were not mentioned. |
| **Wang et al. (2013) [21]** | |
| Method | Randomized, prospective, controlled study |
| Participants | 60 patients with primary tinnitus  Gender: 33 men and 27 women  Mean age: 50 ± 4 years in the treatment group and 51 ± 5 years in the control group.  Tinnitus duration: 2.1 ± 0.1 weeks in the treatment group and 2.2 ± 0.2 weeks in the control group.  Location of tinnitus: not mentioned  Audiography: not mentioned  Groups: treatment group, 30 patients; control group, 30 patients  Inclusion criteria: patients aged 40–75 years, with tinnitus as the chief complaint.  Exclusion criteria: patients were excluded if they (1) had disorders of the external or the middle ear, (2) had an intracranial neoplasm, (3) were taking medicines that might affect the study results, or (4) had severe disorders of other organs, such as vascular malformation, hypertension, hematologic diseases, diabetes mellitus, and mental disorders. |
| Interventions | Treatment group:  (1) EA: four acupoints, which included Tinghui (GB2), Yifeng (SJ17), Ermen (SJ21), and Tinggong (SI19), were selected as the main acupoints. A series of other acupoints were listed as adjunct acupuncture points, such as Baihui (DU20), Shuigu (GB8), Waiguan (SJ5), Zhongzhu (SJ3), Xiaxi (GB43), and Zulinxi (GB41). According to syndrome differentiation by TCM, Hegu (LI4), Yanglinquan (GB34), and Taichong (LR3) were used for excessive syndrome, and Taixi (KI3), Sanyinjiao (SP6), and Zusanli (ST36) were used for deficiency syndrome. In each treatment session, any two of the main acupoints were selected for EA, while the other acupuncture points were manually manipulated. Each patient underwent 10 daily EA sessions for two consecutive courses.  (2) Medication: puerarin injection 400 mg, lidocaine 10 ml, flunarizine 10 mg, intravenous once a day for 15 × 2 sessions.  Control group:  Drug therapy only. |
| Outcomes | Efficacy rate |
| Notes | Loss to follow-up/withdrawal/drop-out rates and adverse events were not mentioned. |
| **Zhang (2002) [22]** | |
| Method | Randomized, prospective, controlled study |
| Participants | 128 patients with primary tinnitus  Gender: 69 men and 59 women  Mean age: 54 years in the treatment group and 53 years in the control group.  Tinnitus duration: 2 days to 2 years in the treatment group and 5 days to 27 months in the control group.  Location of tinnitus: not mentioned  Audiography: not mentioned  Groups: treatment group, 88 patients; control group, 40 patients  Inclusion criteria: patients with tinnitus  Exclusion criteria: not mentioned |
| Interventions | Treatment group:  Tinghui (GB2), Yifeng (SJ17), and Tinggong (SI19) were selected as the main acupoints. When the sensation of Deqi was felt, electrical stimulation with continuous waves was administered to GB2 and SI19. Based on syndrome differentiation according to TCM, manual acupuncture was conducted at Taichong (LR3), Qiuxu (GB40), and Zhongzhu (SJ3) for flaming-up of liver-fire, Hegu (LI4) and Waiguan (SJ5) for disturbance of exogenous wind-heat, Fenglong (ST40), Yinlingquan (SP9), Taichong (LR3), and Qiuxu (GB40) for stagnancy of phlegm-fire, and Taixi (KI3) and Sanyinjiao (SP6) for deficiency of the kidney-yin. Each patient underwent 10 daily sessions of treatment for three consecutive courses.  Control group:  The treatment procedure was the same as in the treatment group, except that the acupuncture needles were manipulated manually. |
| Outcomes | Efficacy rate |
| Notes | Loss to follow-up/withdrawal/drop-out rates and adverse events were not mentioned. |
| **Marks et al. (1984) [23]** | |
| Method | Randomized, double-blind, cross-over controlled trial |
| Participants | Fourteen patients with constant chronic unilateral tinnitus were recruited. After 1 week of measurements, two treatments were administered, 1 week apart. Then, after a 3-week washout period, the procedures were reversed.  Gender: 7 men, 7 women  Age: 25–70 years.  Location of tinnitus: tinnitus in both ears 33/50  Audiography: not mentioned  Groups: treatment (EA) group, 14 patients; control (placebo acupuncture) group, 14 patients  Inclusion criteria: patients with chronic tinnitus and history of treatment with no subjective change.  Exclusion criteria: not mentioned. |
| Interventions | Treatment group:  A combination of acupoints together with an auricular point for vertigo was selected. EA was performed using alternating low (6–10 Hz) and high (100 Hz) frequencies. Each treatment lasted for 20 min.  Control group:  Non-penetrating acupuncture was used as the placebo treatment. The needles were used to prick the skin and were removed immediately without the patients’ knowledge. Patients were not informed about Deqi. |
| Outcomes | Efficacy of treatment evaluated using a verbal description by the patient, tinnitus matching, visual analogue scales of the loudness/severity of tinnitus. Chi-square test with Yates modification and a *t*-test were used for statistical analyses. |
| Notes | Loss of follow-up/withdrawal/drop-out rates and adverse events were not mentioned. |
| **Wang et al. (2010) [24]** | |
| Method | Randomized, single-blind, placebo-controlled design |
| Participants | A total of 60 patients with tinnitus for more than 3 months were recruited. After 4 weeks of follow-up, 50 participants were investigated.  Gender: 46 men, 4 women  Age: 30–70 years; mean ± SEM, 53.12 ± 2.84 years.  Tinnitus duration: more than 3 months; mean ± SEM, 11.86 ± 2.40 years  Location of tinnitus: tinnitus in both ears 33/50  Audiography: hearing loss (42/50)  Matched frequency of ongoing tinnitus: 5218 ± 624 Hz  Reduction in quality of daily life (0–3): 1.86  Groups: treatment (EA) group, 20 patients; control (manual acupuncture) group, 20 patients; placebo group, 20 patients.  Inclusion criteria: patients aged 30–70 years with tinnitus for more than 3 months.  Exclusion criteria: patients with coexisting mental illness or severe heart diseases. |
| Interventions | Treatment group:  Bilateral local points (GB8, TE7, GB2, GB20, and GV20) and bilateral remote points (TE3 and ST36) were selected. Electrical stimulation was applied with a square burst pulse of 0.1 ms duration and an alternative frequency of 2/100 Hz at 3-s intervals. Each patient underwent six daily sessions of 25 min each for 6 consecutive weeks.  Control group:  The treatment procedure was the same as that in the EA group, except that the acupuncture needle was manipulated manually.  Placebo group:  Placebo needles (half-cut, blunt-tip) were used. |
| Outcomes | Visual analogue scale (VAS) was used to assess the outcome in this study. VAS is a psychometric response scale which measures subjective characteristics or attitudes that cannot be directly measured. When responding to a VAS item, respondents specify their level of agreement to a statement by indicating a position along a continuous line between two end points.  Tinnitus frequency on a 5-point scale from 0 to 4, loudness on a 4-point scale from 0 to 3, reduction in quality of life on a 4-point scale from 0 to 3, and an overall evaluation of the treatment effect on a 6-point scale from -2 to 3 was evaluated by the patients at baseline, after-treatment and at 1-month-after. Frequency and intensity of ongoing tinnitus were also assessed using an arbitrary wave form generator. |
| Notes | Sample size calculation indicated that a total of 45 subjects were required to meet the study criteria. |
