# Supplementary material for: Electroacupuncture for Tinnitus: A Systematic Review
Source: PLoS One. 2016 Mar 3;11(3):e0150600. doi: 10.1371/journal.pone.0150600 (PMC4777560; doi:10.1371/journal.pone.0150600)
Supplement: S2 Table — (DOCX) [file pone.0150600.s004.docx]

**S2 table.** Reasons for exclusion of the 11 studies

| **Name** | | **Reason for exclusion** |
| --- | --- | --- |
| Ao et al. (2013) | [25] | No randomization |
| Qing C  (2014) | [26] | No control group |
| Yuan D (2012) | [27] | No control group |
| He et al. (2012) | [28] | Electroacupuncture combined with medicine and acupoint-injection therapy were administered in the intervention group while medicines alone were used in the control group, which did not meet the inclusion criteria. |
| Li et al. (2011) | [29] | No control group |
| Qin (2011) | [30] | No real randomization. |
| Shi and Wei (2012) | [31] | Electroacupuncture combined with auricular seed pressing therapy was administered in the intervention group while medicines were used in the control group, which did not meet the inclusion criteria. |
| Wang (2014) | [32] | Electroacupuncture combined with hyperbaric oxygenation therapy was administered in the intervention group while electroacupuncture was used in the control group, which did not meet the inclusion criteria. |
| Yang and He (2009) | [33] | No control group |
| Yang and Zhang (2009) | [34] | Electroacupuncture combined with acupoint-injection therapy was administered in the intervention group while acupuncture was used in the control group, which did not meet the inclusion criteria. |
| Yang and Zhu (2007) | [35] | No control group |
