# Supplementary material for: Electroacupuncture for Tinnitus: A Systematic Review
Source: PLoS One. 2016 Mar 3;11(3):e0150600. doi: 10.1371/journal.pone.0150600 (PMC4777560; doi:10.1371/journal.pone.0150600)
Supplement: S3 Table — (DOCX) [file pone.0150600.s005.docx]

**S3 table. Risks of bias of included studies**

| **Study** | **Random sequence**  **generation** | **Allocation**  **concealment** | **Patients**  **binding** | **Assessor**  **binding** | **Incomplete outcome**  **data** | **Selective outcome**  **reporting** |
| --- | --- | --- | --- | --- | --- | --- |
| Chen at al  (2013) [20] | U | U | U | U | U | U |
| Wang et al.  (2013) [21] | U | U | H | U | U | U |
| Zhang  (2002) [22] | H | U | U | U | U | U |
| Marks et al.  (1984) [23] | U | U | L | L | U | U |
| Wang et al  (2010) [24] | U | U | H | U | L | U |

Quality assessment based on the Cochrane tools for assessing risk of bias.

Abbreviations: L- low risk of bias, H-high risk of bias, U-Unclear (uncertain risk of bias).
