# Supplementary material for: Electroacupuncture for Tinnitus: A Systematic Review
Source: PLoS One. 2016 Mar 3;11(3):e0150600. doi: 10.1371/journal.pone.0150600 (PMC4777560; doi:10.1371/journal.pone.0150600)
Supplement: S4 Table — (DOCX) [file pone.0150600.s006.docx]

**S4 table. Reasons for the risks of bias assessment**

| **Study** | **Risk of bias** | |  | **Reasons for the risk of assessment** |
| --- | --- | --- | --- | --- |
| Chen at al  (2013) [20] | Random sequence generation | U |  | Method of randomization and allocation was not mentioned in detail. |
|  | Allocation concealment | U |  | Allocation concealment was not mentioned. |
|  | Patients binding | U |  | Patients binding was not mentioned |
|  | Assessor binding | U |  | No information provided. |
|  | Incomplete outcome data | U |  | Insufficient reporting of attrition/exclusions to permit judgement of ‘Low risk’ or ‘High risk’. |
|  | Selective outcome reporting | U |  | The study protocol is not available and it is unclear whether the published reports include all expected outcomes. |
| Wang et al.  (2013) [21] | Random sequence generation | U |  | Method of randomization and allocation was not mentioned in detail. |
|  | Allocation concealment | U |  | Allocation concealment was not mentioned. |
|  | Patients binding | H |  | Although placebo acupuncture was used, it is still unclear whether the patients were blinded to the electroacupuncture and manual acupuncture methods. |
|  | Assessor binding | U |  | No information provided. |
|  | Incomplete outcome data | U |  | Insufficient reporting of attrition/exclusions to permit judgement of ‘Low risk’ or ‘High risk’. |
|  | Selective outcome reporting | U |  | The study protocol is not available and it is unclear whether the published reports include all expected outcomes. |
| Zhang  (2002) [22] | Random sequence generation | H |  | Patients were allocated to treatment or control groups according to the visiting sequence. This method of allocating participants was not truly random |
|  | Allocation concealment | U |  | Allocation was not adequately concealed |
|  | Patients binding | U |  | Patients binding was not mentioned |
|  | Assessor binding | U |  | No information provided. |
|  | Incomplete outcome data | U |  | Insufficient reporting of attrition/exclusions to permit judgement of ‘Low risk’ or ‘High risk’. |
|  | Selective outcome reporting | U |  | The study protocol is not available and it is unclear whether the published reports include all expected outcomes. |
| Marks et al.  (1984) [23] | Random sequence generation | U |  | Authors failed to clearly describe how the participants were allocated |
|  | Allocation concealment | U |  | Allocation concealment was not mentioned. |
|  | Patients binding | L |  | Non-penetrating needles were used as the placebo treatment. |
|  | Assessor binding | L |  | Outcome assessor was blind, and the researchers who knew the whole course of treatment were not involved in the outcome assessment. |
|  | Incomplete outcome data | U |  | Insufficient reporting of attrition/exclusions to permit judgement of ‘Low risk’ or ‘High risk’. |
|  | Selective outcome reporting | U |  | The study protocol is not available and it is unclear whether the published reports include all expected outcomes. |
| Wang et al  (2010) [24] | Random sequence generation | U |  | Method of randomization and allocation was not mentioned in detail. |
|  | Allocation concealment | U |  | Allocation concealment was not mentioned. |
|  | Patients binding | H |  | Placebo acupuncture needles were used. But it is still unclear how the patients were blinded to the electroacupuncture and manual acupuncture methods. |
|  | Assessor binding | U |  | No information provided. |
|  | Incomplete outcome data | L |  | In this trial, a total of 45 subjects were required to limit the risk of type I and type II errors to 5% and 20%. In fact, 60 patients were recruited and randomized. And at the end of follow-up period, 10 patients had withdrawn (16.67% withdrawal rate). Plausible effect size among missing outcomes not enough to have a clinically relevant impact on observed effect size. |
|  | Selective outcome reporting | U |  | The study protocol is not available and it is unclear whether the published reports include all expected outcomes. |

Quality assessment based on the Cochrane tools for assessing risk of bias.

Abbreviations: L- low risk of bias, H-high risk of bias, U-Unclear (uncertain risk of bias).
